# In Reply to: Neurological side effects after SARS-CoV-2 vaccinations require thorough and comprehensive investigations

**DOI:** 10.3325/cmj.2024.65.163

**Published:** 2024-04

**Authors:** Grgur Salai, Ruđer Novak, Đivo Ljubičić, Lovorka Grgurević

**Affiliations:** 1 Department of Pulmonology, Dubrava University Hospital, Zagreb, Croatia; 2 Department of Proteomics, Center for Translational and Clinical Research, School of Medicine, University of Zagreb, Zagreb, Croatia; 3 School of Medicine, University of Zagreb, Zagreb, Croatia; 4 Drago Perović Institute of Anatomy, School of Medicine, University of Zagreb, Zagreb, Croatia; *lovorka.grgurevic@mef.hr*

We thank Dr Finsterer for his comments and the editors for allowing us to briefly respond to the points that have been raised.

In January 2022, we initially published a case report on a 48-year-old woman who presented with muscle fasciculations and visual migraine auras without headaches, which occurred shortly after the first dose of BNT162b2 vaccine (1). Following case publication, we received several e-mails from laypeople who had read our case report, complaining of similar phenomena that occurred shortly after vaccination. In order to further investigate these phenomena, we designed a self-administered survey inquiring about our correspondents’ experiences regarding these phenomena. The results were reported as a short communication (2). In both reports (1,2), we emphasized that no causal connection to the vaccine can be inferred, and we classified these phenomena as adverse events following immunization (AEFI) and not as side effects.

Dr Finsterer raised important comments that he structured in six points, which we would like to systematically clarify:

1. When asked whether they were referred to a neurology specialist, 8 participants answered positively (data not published).

2. We did not investigate which instrumental examinations were conducted. We did not have access to the participants' medical documentation, and the participants were not in our medical care. The lack of objective clinical measurements was reported as a study limitation (2).

3. The median latency from vaccination to symptom onset was 14 (4-36.5) days. We agree with Dr Finsterer that other causes should be considered, as we do not claim any causality between vaccination and these phenomena. However, in the setting of Guillain-Barré syndrome, a history of infections is commonly reported in a range from 3 days to 6 weeks before neurological symptoms (3).

4. The participants were indeed not asked to further clarify which type of aura they experienced. Not asking this question systematically was an omission. However, in the open-type questions, participants with “aura” described visual disturbances.

5. The discrepancy between 11 participants who completed the survey and 10 participants who experienced muscle twitching in the post-vaccination phase lies in the fact that one participant did not consider their symptoms to occur directly after vaccination, but rather after SARS-CoV-2 infection (which occurred after the vaccination). We touched upon this in the discussion section by stating that the impact of SARS-CoV-2 infection might be a confounding variable (2).

6. We agree that the latency from symptom onset to completing the survey was very long. Sampling bias was listed as one of the major study limitations.

To conclude, we would like to emphasize that based on our research no causal connection can be inferred between these phenomena and SARS-CoV-2 vaccination (1,2). Furthermore, we are aware of the major limitations that arise from self-report online surveys (2). The primary point of our short communication was to report that our original case report (1) might not be an isolated event in order to potentially drive further research in this field. Finally, we agree that potential AEFI require thorough and comprehensive investigations, and we enthusiastically wait for systematic evaluation of the reported phenomena.
